# Balance Fatigue Design of Cast Steel Nodes in Tubular Steel Structures

**DOI:** 10.1155/2013/421410

**Published:** 2013-09-12

**Authors:** Libin Wang, Hui Jin, Haiwei Dong, Jing Li

**Affiliations:** ^1^School of Civil Engineering, Nanjing Forestry University, Nanjing 210037, China; ^2^Jiangsu Key Laboratory of Engineering Mechanics, Southeast University, Nanjing 210096, China

## Abstract

Cast steel nodes are being increasingly popular in steel structure joint application as their advanced mechanical performances and flexible forms. This kind of joints improves the structural antifatigue capability observably and is expected to be widely used in the structures with fatigue loadings. Cast steel node joint consists of two parts: casting itself and the welds between the node and the steel member. The fatigue resistances of these two parts are very different; the experiment results showed very clearly that the fatigue behavior was governed by the welds in all tested configurations. This paper focuses on the balance fatigue design of these two parts in a cast steel node joint using fracture mechanics and FEM. The defects in castings are simulated by cracks conservatively. The final crack size is decided by the minimum of 90% of the wall thickness and the value deduced by fracture toughness. The allowable initial crack size could be obtained through the integral of Paris equation when the crack propagation life is considered equal to the weld fatigue life; therefore, the two parts in a cast steel node joint will have a balance fatigue life.

## 1. Introduction 

**Figure 1:**
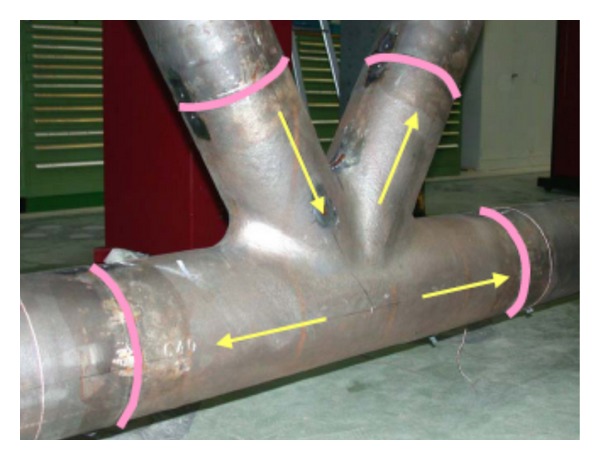
Cast steel node (Pictures are from http://icom.epfl.ch/).

**Figure 2:**
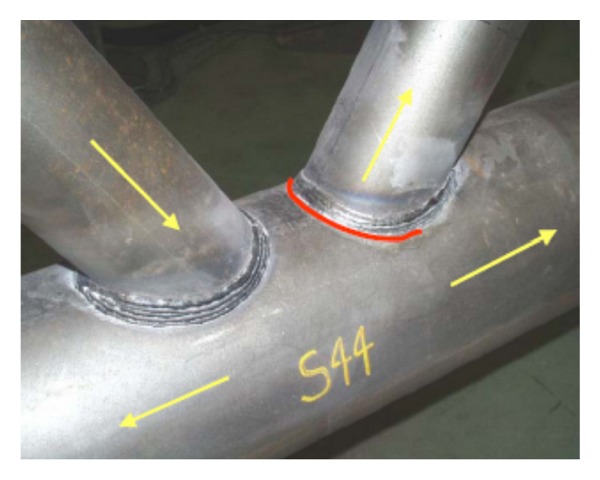
Welded joint (Pictures are from http://icom.epfl.ch/).

Due to their advanced mechanical performance, beautiful appearance, and smooth transitions, flexible forms that multiple steel tubes can intersect from different directions, cast steel nodes are especially applicable to joints of steel structures which are in the state of three-dimensional stress in civil engineering [1, 2]. The cast steel nodes were first introduced to the offshore structures in the 1980s [3]. From then on, many countries start using cast steel nodes in the design and construction of offshore structures. Numerous studies have been carried out on cast steel nodes in ocean engineering, and there are some reference standards now [4]. Nowadays, cast steel nodes also have been widely applied in civil engineering owning to its unique advantages. Compared with the traditional welded joints, cast steel nodes have some obvious advantages (see in Figures 1 and 2). (1) The girth butt welds are used which are perpendicular to the axis of the tube, so that the welding secondary stress can be reduced greatly. (2) The welded seams are far from the node core area to reduce the weld stress. (3) By chamfering at tube intersections, the stress concentration is reduced. (4) The casting production process has great applicability and can meet various requirements of appearance and mechanics. (5) Cast steel node is good at fatigue resistance and corrosion resistance. 

The laboratory of ICOM in Switzerland did fatigue tests on large-scale steel tube trusses containing cast steel nodes. They studied the fatigue properties of the cast steel nodes and the whole trusses, the influence of casting defects, and compared the fatigue properties of cast steel node with that of traditional welded node [5–9]. The researches in Germany studied and tested the fatigue strength of the girth butt welds in cast steel node [10]. Jin et al. studied the fatigue strength of castings and the girth butt welds in cast steel nodes in China [11–14].

The fatigue capacity of cast steel nodes is decided by two major parts: the casting itself and the girth butt welds between the casting and the steel members. The fatigue resistances of these two parts are very different; the experiment results showed very clearly that the fatigue behavior was governed by the welds in all tested configurations [7, 9]. So, this kind of joint contains two greatly unbalanced parts from an overall fatigue perspective. The balance fatigue design can realize an optimization of performance and economy.

The casting defects are unavoidable in the process of casting. If defects are permitted, there must remarkably influence the fatigue property of casting part, as cracks are initiated from defects. If defects are not permitted, there will be lots of fabrication difficulties, more restrictions on the node form, and higher probabilities of unacceptable quality production. Moreover, the additional fatigue strength of the casting material exceeding the welds is wasted. The key point of balance design is to calculate the allowable initial defect size and make the casting fatigue strength containing defects coordinated with the weld fatigue strength. Thus, it could reach a best economic efficiency by optimum using of the materials. The damage tolerance method is used in this paper to quantitatively calculate the allowable initial defects size in castings for a balance fatigue design.

## 2. Method of Damage Tolerance Design

Method of damage tolerance design is based on the fracture mechanics theory, nondestructive test, and fracture toughness test, which is firstly applied in the fatigue balance design of cast steel node by Nussbaumer and Haldimann-Sturm [7, 9].

### 2.1. Stress Intensity Factor

In fracture mechanics, the stress intensity factor (SIF) expression is

(1) $$\begin{array}{c} K=f\sigma \sqrt{\pi a}, \end{array}$$

in which *f* is correction factor decided by the crack shape, crack location, and loading mode. *f* may be a constant as it is proposed in [7, 9], or in function of the crack size along different possible crack propagation directions as it is fitted in this paper by FEM simulation results.

### 2.2. Fatigue Crack Growth Rate

Fatigue crack growth rate can be expressed by Paris-Erdogan equation:

(2) $$\begin{array}{c} \frac{\text{d}a}{\text{d}N}=C{\left(\Delta K\right)}^{m}, \end{array}$$

in which Δ*K* is SIF range, Δ*K* = *K*_max⁡_ − *K*_min⁡_; *C*,  *m* are material constants.

### 2.3. Estimation of Residual Life

#### 2.3.1. Initial Crack Size *a*_0_

The initial crack size has great influence on the fatigue life and should be prudently decided [15]. The casting defects are unavoidable in the process of casting like gas holes, slack inclusions, or shrinkages. These defects can be modeled as two-dimensional cracks, and this is a conservative assumption [7, 9]. There are two kinds of normalized cracks in castings: surface cracks and inner cracks. The key point of balance design is to calculate the allowable initial defect size *a*_0_, making the casting fatigue strength containing defects coordinated with the weld fatigue strength.

The real initial crack size *a*_0_′ can either be evaluated by casting process numeral simulation or be detected by nondestructive testing. If *a*_0_′ is smaller than the assessed allowable initial defect size *a*_0_, the fatigue life is still governed by the weld.

#### 2.3.2. Critical Crack Size *a*_*c*_

Critical crack size is the maximum size allowed under certain stress condition, in the case of brittle failure. It is often expressed by *a*_*c*_, and determined by the fracture toughness *K*_*Ic*_:

(3) $$\begin{array}{c} a_{c}=\frac{1}{\pi }{\left(\frac{K_{Ic}}{f\sigma }\right)}^{2}\;\;\left(\text{mm}\right). \end{array}$$

The final crack size for castings is decided by the minimum of 90% of the wall thickness as a through-thickness crack and the value calculated by fracture toughness (see (3)).

#### 2.3.3. Fatigue Crack Propagation Life

The fatigue crack propagation life is deduced through the integral of the Paris-Erdogan equation as the constant-amplitude stress is considered:

(4) $$\begin{array}{c} N_{P}=\int_{N_{0}}^{N_{f}}\text{d}N=\int_{a_{0}}^{a_{c}}\frac{\text{d}a}{C{\left(\Delta K\right)}^{m}}=\int_{a_{0}}^{a_{c}}\frac{\text{d}a}{C{\left(f\Delta \sigma \sqrt{\pi a}\right)}^{m}}. \end{array}$$

If *f* is the function of *a*, the (*a*_*c*_ − *a*_0_) can be divided into several intervals,  *f* and *N*_*pi*_ can be calculated with the mean value of *a* in each interval. Then, by summing the *N*_*pi*_ of each interval, the fatigue crack propagation life can be evaluated.

## 3. Balance Fatigue Design of Cast Steel Nodes

Because of the defects existing, the fatigue mechanical performance of castings is lowered. The defect size and location can be detected by nondestructive testing method after casting fabrication. So, the key point is to investigate and evaluate the influence degree and how much the residual strength is. 

This section focuses on the design process of cast steel nodes with initial defects, proposing the method of optimization and balance design. The main procedures are as follows.

(1)   *The Node Shape Design.* The node shape can be designed preliminarily according to the mechanics requirements from the whole structure force-bearing demand. and then may be modified according to the pouring and casting process technical feasibility.

(2)  *Optimization Design of the Girth Butt Welds.* The fatigue capability of the joint is governed by the welds, so the girth butt welds should be designed optimally. The hot spot stresses should be analyzed on different weld details, and the optimum weld design detail can be decided by the hot stress life curve; therefore, the weld life *N* can be calculated as the whole joint life which will be used to evaluate the allowable initial crack size *a*_0_.

(3)  *Casting Quality Level Determination. *Higher casting quality cost more; there will be lots of fabrication difficulties, more restrictions on the node form, and higher probabilities of unacceptable quality production. Moreover, the additional fatigue strength of the casting material exceeding the welds is wasted, so reasonable casting quality requirements can balance the strengths of the two parts in one joint. According to the joint fatigue life *N*, the stress amplitude in the casting part, and the material *S-N *curves at different cast quality level, the reasonable casting quality levels can be determined.

(4)  *Real Initial Crack Size Determination. *During the design phase, the real initial crack size *a*_0_′ can be evaluated by pouring and casting process numeral simulation through some professional software.

(5)  *Justification of a*_0_′. The casting defects can be modeled as cracks and an equivalent initial crack size *a*_0_ represents the initial defect size. The determination of an allowable *a*_0_ value can be deduced by fracture mechanics, when the critical crack size *a*_*c*_ is determined and the life is equal to the joint life *N*. Then, the real Initial crack size *a*_0_′ is compared with *a*_0_; if it is greater than the allowable size, the design should be modified.

**Figure 3:**
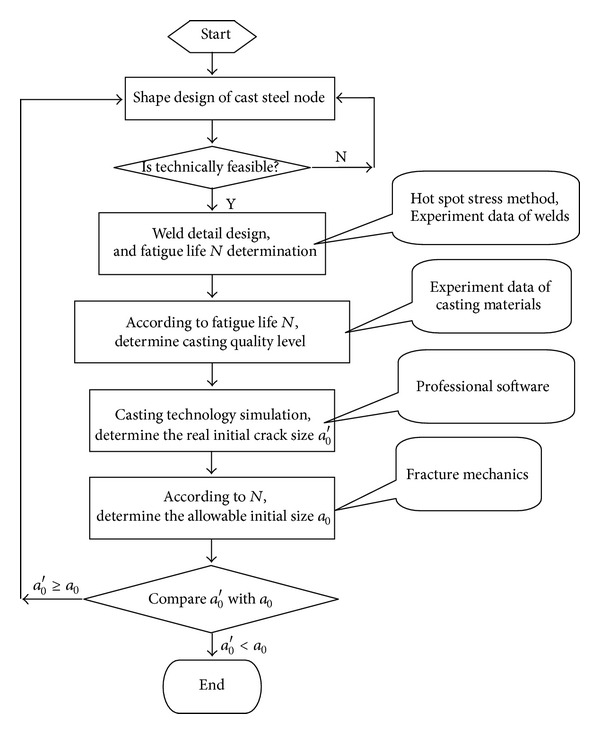
Procedures of optimization design of cast steel nodes.

The procedures previously mentioned provide a basis for balance design and production processes of cast steel node, and the flowchart is shown in Figure 3.

## 4. Determination of Allowable Initial Size *a*_0_

Nussbaumer and Haldimann-Sturm proposed the correction factor *f* is constant [7, 9]; in this study it is found a variable not only in function of *a*, but also in function of propagation direction:

(5) $$\begin{array}{c} K_{i}=f_{i}\left(\frac{a}{t}\right)\sigma \sqrt{\pi a}, \end{array}$$

in which *K*_*i*_ is SIF along propagation direction *i*. The crack is modeled by FEM, ANSYS software, and *f*_*i*_ is fitted to get the *K*_*i*_ expression.

The SIF range is simulated by FEM

(6) $$\begin{array}{c} \Delta K_{I}\left(a\right)=f\cdot \Delta \sigma \cdot \sqrt{\pi \cdot a}, \end{array}$$

in which Δ*σ* = *σ*_max⁡_ − *σ*_min⁡_. 

**Figure 4:**
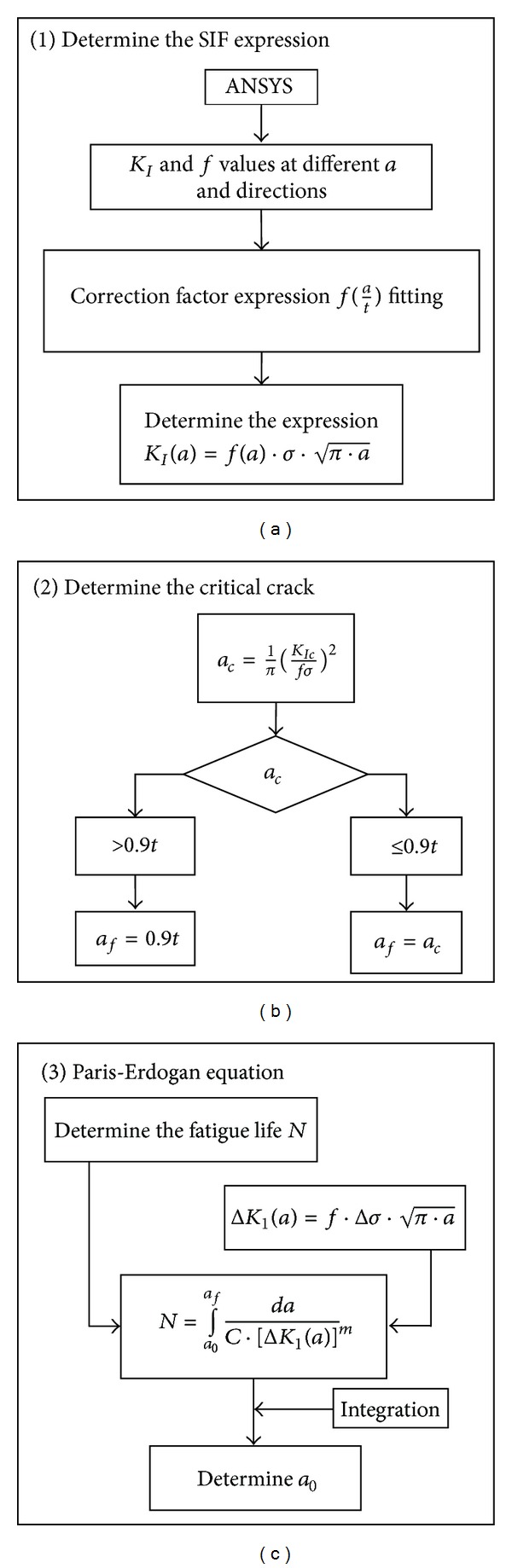
Calculation of initial crack size.

Determination of allowable initial defect size *a*_0_ is in Figure 4.

## 5. Examples

The design of cast steel node in tour tower steel structure on the platform of Hangzhou Bay Bridge is demonstrated to show the balance design procedures.

**Figure 5:**
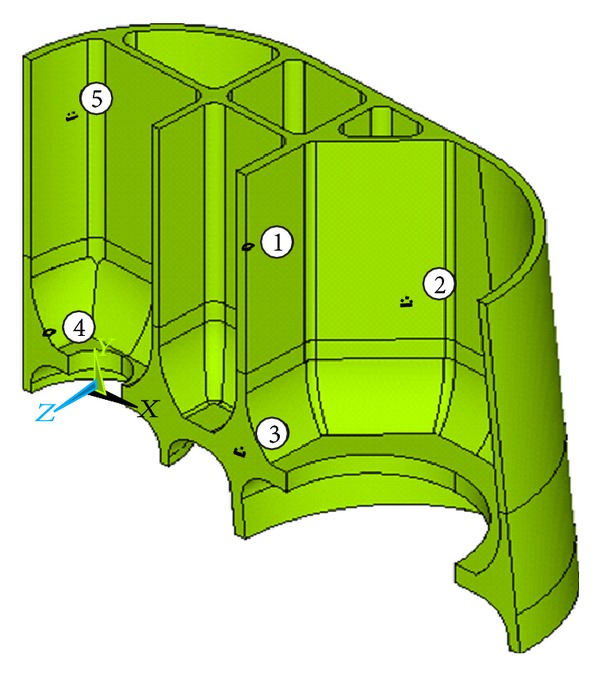
Node model and defect locations.

Five typical defects were found in the castings, in which *②*, *③*, and *⑤* are modeled as inner cracks and *①*, *④* are modeled as surface cracks (Figure 5). Due to the longitudinal symmetry, half model is analyzed; the cracks are modeled as a half of circular crack and a quarter of circular crack. The defect locations are shown in Figure 5. The designed real initial crack size *a*_0_′ are listed in Table 1.

### 5.1. Modeling the Cracks

**Figure 6:**
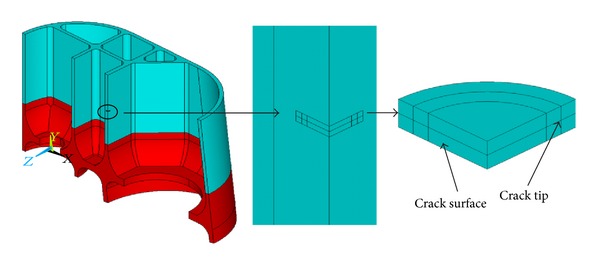
Surface crack body.

**Figure 7:**
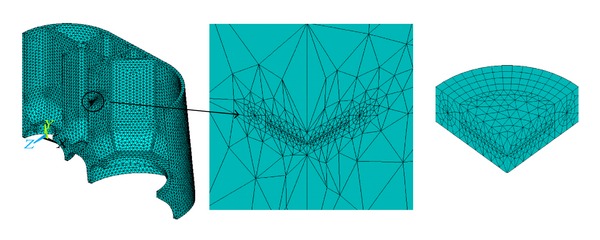
Refined surface crack body meshes.

**Figure 8:**
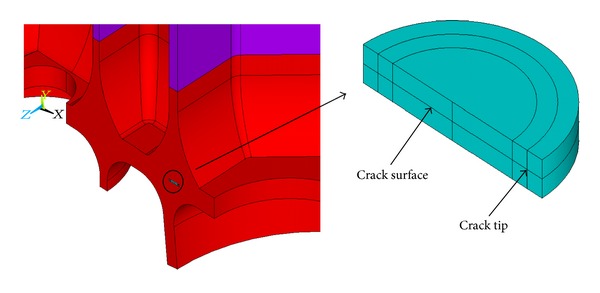
Inner crack body.

**Figure 9:**
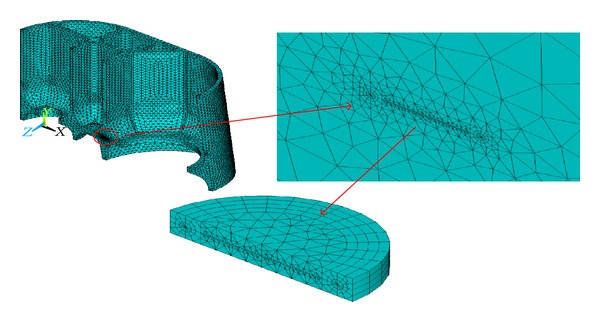
Refined inner crack body meshes.

The surface crack body of defect *①* is modeled by a quarter of one thin circle cylinder due to the symmetry (Figure 6), consisting of two parts: upper part and lower part to construct the crack surface and crack tip. The crack body meshes are refined (Figure 7). The inner crack body of defect *③* is modeled by a half of one thin circle cylinder (Figure 8), also consisting of two parts: upper part and lower part to construct the crack surface and crack tip. The crack body meshes are refined (Figure 9).

### 5.2. Cyclic Loadings

**Figure 10:**
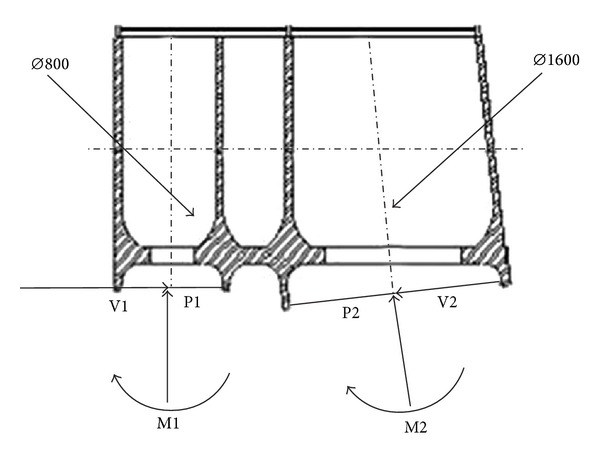
Loads on node.

The constant-amplitude cyclic loadings inducing fatigue on the node are shown in Figure 10, Table 2, consisting of axis force *P*, sheering force *V*, and bending moment *M* [16].

### 5.3. Calculation of Allowable Crack Size

Defects *①* and *③* are chosen to demonstrate the calculation procedures of allowable initial crack size of surface and inner defect.

#### 5.3.1. Calculation of Allowable Initial Surface Crack Size of Defect *①*

**Figure 11:**
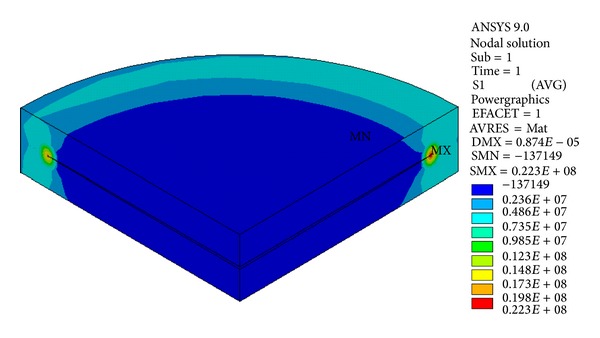
Principal stress contour of crack body *①*.

The principal stress contour of the crack body is shown in Figure 11.

**Figure 12:**
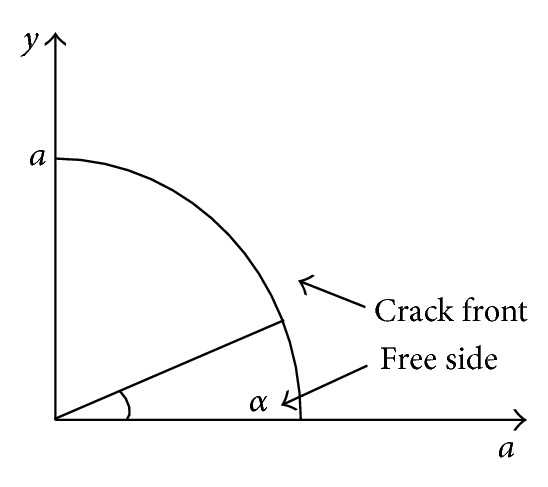
Prorogation directions.

Defect *①* locates at the edge of inner stiffener plate of the node, where the node wall thickness is *t* = 45 mm. Five cases of different crack depth are analyzed by ANSYS, that is, *a* = 15 mm, 20 mm, 25 mm, 30 mm, and 35 mm in each case the crack body is meshed along sixteen prorogation directions as symbol *α*, that is, *α* = 0°, 6°, … , 90°. See Figure 12.

**Figure 13:**
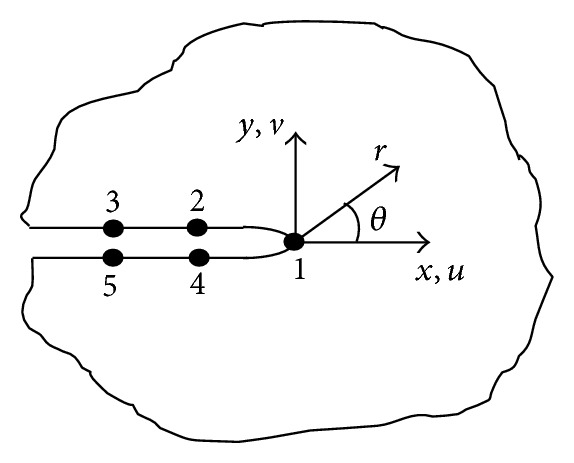
Crack path defined in ANSYS.

In ANSYS, the crack propagating path should be defined, a crack path is defined by nodes 1, 2, 3, 4, 5, for instance (Figure 13). The crack SIF is calculated as the average of SIF values at the nodes on the crack path.

The expression of *K*_*I*_ is [17]

(7) $$\begin{array}{c} K_{I}=\frac{E^{\prime }}{4}\sqrt{\frac{\pi }{2L}}\left[4\left(\nu_{2}+\nu_{4}\right)-\nu_{3}-\nu_{5}\right], \end{array}$$

In which *E*′ = *E*/(1 − *μ* ^2^)   (plane strain), *E* is elastic modulus, *μ* is passion ratio, *ν* is node displacement on the crack path, and *L* is the length of the crack tip element.

**Figure 14:**
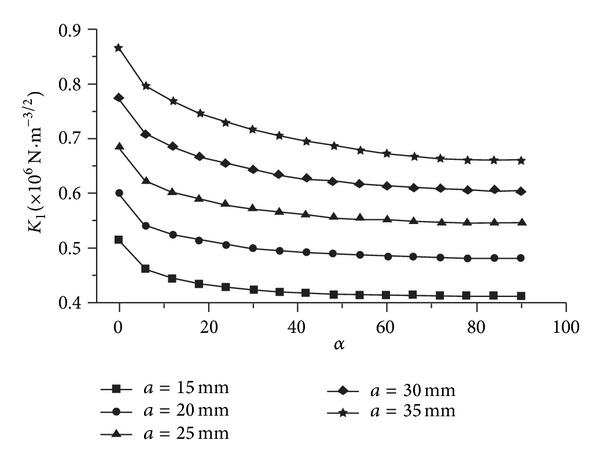
SIF fitting of defect *①*.

SIFs of five cases along sixteen direction path are shown in Figure 14, and a curve is fitted for each case to show the trend. The SIF values are obviously not constant; their changing ratio ranges are 63% ~ 67% along the direction from *α* = 0° to *α* = 90° and 26% ~ 410% along the crack depth from *a* = 15 mm to *a* = 35 mm.

**Figure 15:**
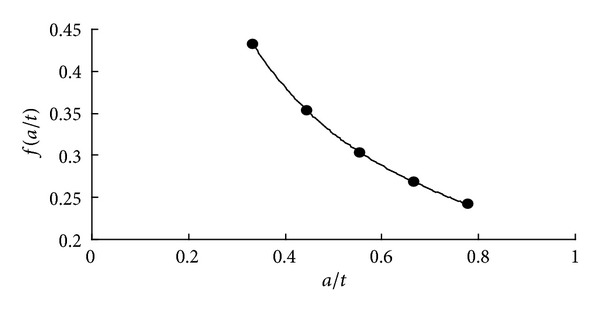
Fitting curve of *f* at the path of *α* = 0°  .

The path of maximum SIF value is considered where *α* = 0° (see in Figure 14). Along the path of *α* = 0°, the values of *f* at five crack depths are calculated through (5), and a quartic polynomial is fitted (Figure 15). 

The expression of quartic polynomial is

(8) f(at)=1.1486(at)4−3.7431(at)3 +4.7291(at)2−2.9461(at)+1.0127.

By substitution of the data of wall thickness *t* = 45 mm, the expression of *K* in function of *a* can be obtained through *f* fitting,

(9) KI=σπ (280103.6427a4.5−41076.5432a3.5   +2335.3580a2.5−65.4689a1.5  +1.0127a0.5).

The casting material used in the steel tower structure at Hangzhou Bay is GS-20Mn5V, and there is no fatigue and fracture data available for reference. As the element compositions and mechanical properties of GS-Mn5V are similar to ZG20SiMn (Chinese Steel Grade) [18], the material parameters of ZG20SiMn in [19] are used approximately: *C* = 2.25510^−13^, *m* = 3.9917. The critical *J* integral of ZG20SiMn is *J* = 102.6  (N/mm), so the fracture toughness can be deduced in the case of plane strain

(10) $$\begin{array}{c} K_{Ic}=\sqrt{J\cdot \frac{E}{1-\mu^{2}}}=152.4\left(\text{MP}\sqrt{m}\right). \end{array}$$

The allowable initial crack size of defect *①* is calculated following the flowchart (Figure 4) with the FEM analysis results; the result is *a*_0_ = 27.6 mm, and the designed real crack size is *a*_0_′ = 15.4 mm, so the casting strength is satisfied.

#### 5.3.2. Calculation of Allowable Initial Inner Crack Size of Defect *③*

**Figure 16:**
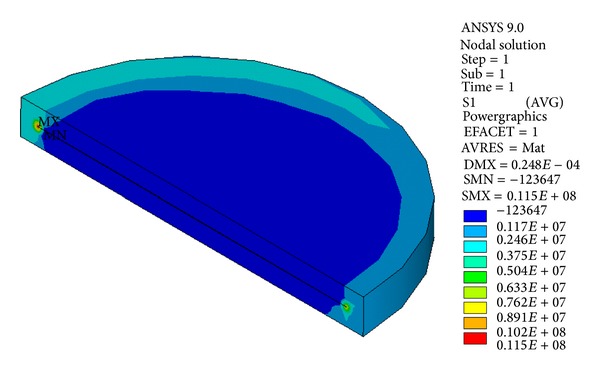
Principal stress contour of crack body *③*.

The principal stress contour of the crack body is shown in Figure 16.

**Figure 17:**
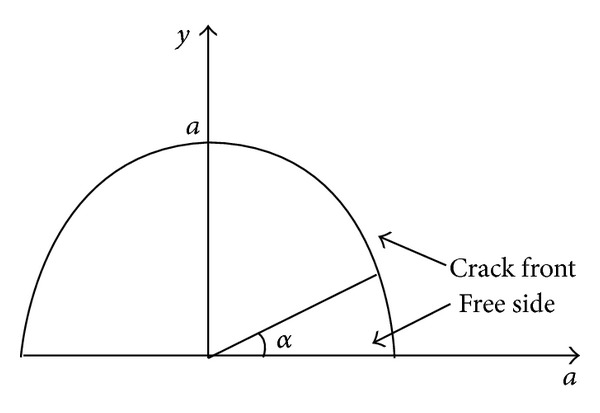
Prorogation directions.

Defect *③* locates at the inner of the node, where the horizontal node thickness along the crack propagation direction is *t* = 800 mm. Five cases of different crack depth are analyzed by ANSYS, that is, *a* = 25 mm, 30 mm, 35 mm, 40 mm, and 45 mm. In each case, the crack body is meshed along thirteen prorogation directions as symbol *α*, that is, *α* = 0°, 15°, …, 180°. See Figure 17.

**Figure 18:**
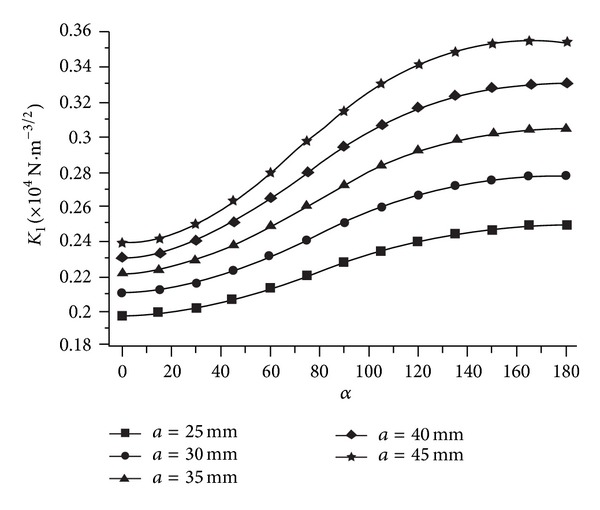
SIF fitting of defect *③*.

SIF values of five cases along thirteen direction paths are shown in Figure 18, and a curve is fitted for each case to show the trend. The SIF value changing ratio ranges are 21% ~ 44% along the direction from *α* = 0° to *α* = 180°, and 23% ~ 47% along the crack depth from *a* = 25 mm to *a* = 45 mm.

**Figure 19:**
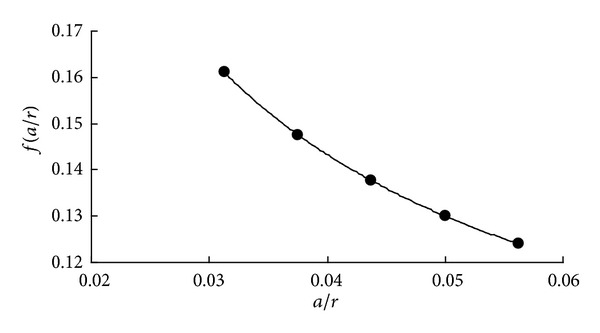
Fitting curve of *f*at the path of *α* = 180°.

The path of maximum SIF value is considered where *α* = 180° (see in Figure 18). The fitting quartic polynomials are shown in Figure 19.

One has

(11) f(at)=30037(at)4−5905.1(at)3 +457.07(at)2−17.529(at)+0.4142.

By substitution of the data of thickness *t* = 800 mm, the expression of *K* in function of *a* is

(12) KI=σπ(73332.5195a4.5−11533.4a3.5+714.2a2.5−21.9a1.5+0.4142a0.5);

the allowable initial crack size of defect *③* is calculated following the flowchart (Figure 4) with the FEM analysis results, the result is *a*_0_ = 221.3 mm, and the designed real crack size is *a*_0_′ = 29.5 mm, so the casting strength is satisfied.

#### 5.3.3. Results of Five Defects

The results of five defects are listed in Table 3, the ratio ranges of allowable initial defect size to wall thickness are from 28% to 67%, the max-ratio happens at the thinnest wall thickness location, and the min-ratio happens at the thickest inner node thickness location. The minimum allowable initial defect locates at defect *③*, and the maximum allowable defect locates at defect *②*.

It is shown that the present design with five defects satisfies the strength demand, and the results and conclusions can be referenced for further design improvement.

## 6. Conclusions

This paper focuses on the balance fatigue design of the two parts in a cast steel node joint using fracture mechanics and FEM; the key point in the procedures is how to calculate the allowable initial equivalent crack size; the following main conclusions can be drawn.

1. The allowable existence of reasonable initial defects make the castings fatigue strength coordinated with the weld fatigue strength to realize a balance design avoiding the material strength waste.
2. Casting defects can be equivalently treated as cracks and simulated by FEM software fracture module.
3. The engineering example shows the ratio of allowable initial defect size to node thickness ranges from 28% to 67%, in which the high ratio may not be allowed without this balance design concept.
4. The crack SIF values shown in the fracture mechanic simulation results are obviously not constant; it depends on the crack depth and crack propagation direction and their changing ratio can be up to 410%; the SIF accuracy will seriously affect the fracture results, so whether the SIF can be simplified as a constant correction factor or not should be determined carefully by a given requisite degree of accuracy.
5. The node has distinct different fatigue capabilities under some influences, for example, locations, directions, and loading conditions. For the fatigue sensitive ones, it is important to estimate the defect propagating direction and the range of SIF Δ*K* correctly.

## Figures and Tables

**Table 1:** Real initial defect size *a*_0_′ (mm).

Defect	*①*	*②*	*③*	*④*	*⑤*
*a*_0_′	15.4	7.6	29.5	21.6	10.8

**Table 2:** Constant amplitude fatigue loads.

	Load case		P (KN)	V (KN)	M (kN · m)
On Tube of Ø 800	Case 1	Max	756.391	24.132	15.5521
	Case 2	Min	−736.617	−23.456	−15.1126
On Tube of Ø 1600	Case 1	Max	2000.222	49.266	209.1114
	Case 2	Min	−1956.73	−48.601	−214.845

**Table 3:** Results of five defects.

Defect	Crack path	Wall thickness *t* (mm)	*α*_0_ (mm)	*α*_0_′ (mm)	*α*_0_/*t* (%)
*①*	*α* = 0°	45	27.6	15.4	61
*②*	*α* = 90°	35	23.3	7.6	67
*③*	*α* = 180°	800	221.3	29.5	28
*④*	*α* = 0°	86	37	21.6	43
*⑤*	*α* = 180°	45	17.9	10.8	40
